# Evaluating systematic reviews in urology: A practical guide

**DOI:** 10.4103/0970-1591.33441

**Published:** 2007

**Authors:** Prathap Tharyan

**Affiliations:** Department of Psychiatry, South Asian Cochrane Network, Prof. BV Moses Centre for Clinical Trials and Evidence-Based Medicine, Christian Medical College, Vellore - 632 002, Tamil Nadu, India

**Keywords:** Meta-analysis, systematic review

## Abstract

Systematic reviews of good quality randomized controlled trials that have little heterogeneity (variability) are considered to provide the best source of evidence for the efficacy of interventions in healthcare. With the recent national provision for access to *The Cochrane Library* to all residents in India, urologists and other clinicians now have access to this reliable source of regularly updated systematic reviews. This article uses six systematic reviews relevant to urologists from *The Cochrane Library* produced by different collaborative review groups in *The Cochrane Collaboration* to illustrate the methods used to minimize bias, improve transparency and provide reliable estimates of treatment effects. Issues in evaluating results, especially when subsequent trials produce discrepant results, are discussed.

The busy urologist wishing to practice evidence-based urology needs a reliable source of evidence to incorporate into his/her clinical expertise and the values of his/her patients. Well-conducted and reported randomized controlled clinical trials provide the essential building blocks of reliable evidence for the effects of interventions, since they minimize bias, confounders and the effects of chance or coincidence.[1,2] However, the results of a single trial only rarely provide reliable estimates of effectiveness that are generalizable to multiple settings, partly because they may be too small to reveal significant differences between interventions or because they may be too focused on a particular type of patient to provide a result that can be either easily or reliably generalized to future patients or patients in other settings.[3] In addition, the amount of information about healthcare, including that from randomized trials, is now overwhelming.[4] Urologists and other clinicians, need high quality information, though much of what is available is of poor quality.[5] Consequently, vast resources are wasted each year on healthcare that is not effective and may even be harmful.

## SYNTHESIZING EVIDENCE FROM TRIALS OF INTERVENTIONS

Clinicians have always used review articles as sources of evidence; unfortunately, empirical studies have shown that traditional, ‘narrative’ review articles, even when written by experts, tend to be biased and of variable (often poor) quality, because they do not usually have a methodology section that describes in a transparent and reproducible manner the methods and databases used to search for trials, include or exclude trials, extract data and assess quality of included trials.[4] They also usually use the method of ‘vote-counting’, where each trial is assigned equal weight in the overall analysis of effects, irrespective of trial size or quality, leading to erroneous or inconclusive results.[1,3,4]

Systematic reviews use explicitly described methods to limit bias in the formulation of an answerable question about the efficacy and safety of an intervention, the search and retrieval of trials, selection of relevant trials according to pre-stated criteria, assessment of quality and exclusion of poor quality trials (or evaluating the effects of including such trials on the overall evidence). Where data permit, they use the statistical technique of meta-analysis, where the effect estimates from individual trials (odds ratios, relative risks, risk differences for binary outcomes and mean difference for continuous outcomes) along with measures of uncertainty and precision (95% or 99% confidence intervals) are statistically pooled together with differential weighting of trials, where trials with larger sample sizes, more events of interest and less variance, contribute more to the overall evidence. The weighted pooled results offer more reliable estimates of the effect of relevant findings from all trials on a particular topic and not just the results of a few, readily available studies. Methods to assess and deal with heterogeneity (variability) in study results enable interpretation of the appropriateness of pooled results. These results can then be used to establish whether scientific findings are consistent and generalizable across populations, settings and treatment variations or whether findings vary significantly by particular subgroups.[1,3,4,6]

For these reasons, systematic reviews of good quality randomized controlled trials, with large sample sizes and with little variation in results (heterogeneity), offer the best available evidence of treatment effects and are considered to be evidence of the highest level in the hierarchy of research designs evaluating effectiveness of interventions.[1,7]

## SYSTEMATIC REVIEWS AND THE COCHRANE COLLABORATION

*The Cochrane Collaboration* (www.cochrane.org) is the world's largest organization dedicated to preparing, maintaining and promoting the accessibility of systematic reviews of the effects of healthcare interventions. These reviews are published in electronic form in The Cochrane Library (www.thecochranelibrary.com), a collection of seven databases that include the Cochrane Database of Systematic Reviews that currently (Issue 1 2007) contains 4655 regularly updated systematic reviews and protocols of reviews in preparation. Also available are abstracts of systematic reviews in healthcare published elsewhere in the Database of Reviews of Effectiveness (DARE). The overall quality of Cochrane systematic reviews has been judged to be of greater methodological rigor than systematic reviews published in printed journals indexed in MEDLINE[8] and less biased in interpretation of results than systematic reviews sponsored by industry sources.[9] Another useful database is The Cochrane Controlled Trials Register (CENTRAL) that currently contains references, mostly with abstracts, of more than 4,89,000 controlled clinical trials—easily the largest collection of such trials in the world.

Urologists (and all residents) in India now have complimentary access to the full contents of The Cochrane Library, thanks to sponsorship provided by the Indian Council of Medical Research (ICMR) that recently signed a three-year contract for a national subscription with the publishers, John Wiley and Sons. The full potential of this far-sighted initiative of the ICMR to improve the quality of healthcare in the country requires clinicians to understand how to assess systematic reviews for methodological quality, relevance and applicability to one's clinical practice. This article uses examples of systematic reviews relevant to urology from the Cochrane Library to develop these themes.

## COCHRANE SYSTEMATIC REVIEWS IN UROLOGY

Systematic reviews of particular interest to urologists in the *Cochrane Database of Systematic Reviews* can primarily be found in those prepared by review authors of the Cochrane Renal Group, the Cochrane Prostatic Diseases and Urologic Cancers Group and the Cochrane Incontinence Group. Examples from these groups shall be used for illustrative purposes: estrogens for incontinence;[10] interventions for primary vesicoureteric reflux;[11] adjuvant chemotherapy for invasive bladder cancer (that used individual patient data);[12] screening for prostate cancer;[13] and Serenoa Repens for benign prostatic hyperplasia.[14] The last shall be discussed in the light of a subsequent randomized controlled trial, Saw Palmetto for benign prostatic hyperplasia[15] that reported contradictory results. An example of a review of relevance to urologists prepared by the Cochrane Schizophrenia Group on the management of sexual dysfunction due to antipsychotic drug therapy[16] shall also be used.

The quality of a systematic review can be assessed by reference to the quality of reporting of meta-analyses (QUOROM) statement that was developed to indicate whether the results of the review were unbiased.[17] Each of these systematic reviews was evaluated against the quality measures in this statement [Table 1]. In the discussion that follows, individual qualities of a systematic review that contribute to its overall status as the ‘gold standard’ for evidence of efficacy will be highlighted with reference to these sample reviews, the QUORUM statement and the Cochrane Collaboration's handbook for systematic reviews of interventions.[18]

**Table 1:** Quality of selected systematic reviews of relevance to urology

QUORUM Checklist items	Estrogens for incontinence in women[10]	Interventions for vesicoureteric reflux[11]	Adjuvant Chemotherapy for invasive bladder cancer[12]	Screening for prostactic cancer[13]	Serenoa repens for benign prostactic hyperplasia[14]	Anti psychotic-induced sexual dysfunction[16]
Structured abstract	Adequate	Adequate	Adequate	Adequate	Adequate; 2002 update of 1998 review	Adequate
Introduction	Adequate	Adequate	Adequate	Adequate	Adequate	Adequate
Methods: search	Specialized register; no language restrictions	Specialized register; no language restrictions; unpublished trials included	Multiple sources; individual patient data; unpublished trials included; language restrictions unclear	Specialized register; other sources; unpublished trials included; no language restrictions	Multiple sources; unpublished trials sought; no language restrictions	Multiple sources; unpublished trials sought; no language restrictions
Methods: selection	PICO stated; primary outcome(s) not clearly stated	PICO stated; primary outcome(s) not clearly stated	PICO stated; primary outcomes stated	PICO stated; primary outcomes stated	PICO stated; primary outcomes stated	PICO stated; primary outcomes stated
Methods: validity	Study quality assessed	Study quality assessed	Data quality assessed and checked	Study quality assessed	Study quality assessed	Study quality assessed
Methods: data abstraction	Independently done	Independently done	Independently done	Independently done	Independently done	Independently done
Methods: study characteristics	Described adequately	Described adequately	Described adequately	Described adequately	Described adequately	Described adequately
Methods: data synthesis	Fixed effects meta-analysis; heterogeneity assessed; subgroups pre-stated; publication bias not assessed	Fixed effects meta-analysis; heterogeneity assessed; subgroups pre-stated; sensitivity analysis planned	Fixed effects individual patient meta-analysis; heterogeneity assessed; subgroups pre-stated; sensitivity analysis done	Fixed and random effects meta-analysis; heterogeneity assessed; subgroups pre-stated;	Random effects meta-analysis; heterogeneity assessed; subgroup and sensitivity analyses pre-stated	Fixed effects meta-analysis; heterogeneity assessments planned; no subgroups planned; publication bias addressed
Results: Trial flow	Described in text	Described in text	Described in text	Described in text	Described in text	Described in text
Results: data synthesis	Adequate	Adequate	Adequate	Adequate; intention to treat used	Adequate; intention to treat used	Adequate; intention to treat used
Discussion	Mostly adequate	Adequate; reader's comments addressed	Adequate; ongoing trial results awaited	Adequate; two ongoing trial results awaited	Adequate	Adequate

QUORUM: Quality of reporting of meta-analysis, PICO= Patients, interventions, comparisons, outcomes

## ASSESSING THE QUALITY OF COCHRANE SYSTEMATIC REVIEWS

The six selected reviews fulfilled criteria laid out in the QOURUM statement for quality, reflecting the uniform methods used across all collaborative review groups in the collaboration to minimize bias and ensure transparency and reproducibility.

### Structured abstract

All the selected reviews had structured abstracts of the background, aims, methods, results and conclusions of the review. In addition, all the reviews also had a plain language summary using non-technical terms to inform consumers and non-clinicians of the results of the review. For example, the plain language summary for the review on management of sexual dysfunction due to antipsychotic medication states: “Drugs commonly used to treat schizophrenia often cause sexual problems. Strategies to manage these problems are additional drugs, short drug holidays, reduction of dose and switching to another drug. In this review we found two pioneering studies but both were small and short. There is some evidence that sildenafil may be an effective drug to add to standard care for treating men with antipsychotic-induced erectile disorder. However before confident claims can be made about this drug many more trials evaluating the several strategies should be undertaken”.[16]

### Introduction

The background sections of these reviews state the reasons why the review was undertaken and included descriptions of the healthcare condition, the magnitude of the problem, the available interventions and areas of uncertainty. If a previous systematic review exists, then the background needs to justify the need for another systematic review. The adjuvant chemotherapy for bladder cancer review[12] noted the presence of prior systematic reviews and remarked on the poor quality and inappropriate statistics used in the included trials; the authors therefore chose to undertake an individual patient data (IPD) meta-analysis, where data from individual patients rather than aggregate data from trials, was included in a meta-analysis. They hoped that with IPD, they would have the ability to carry out detailed data checking and conduct intention-to-treat analysis using appropriate statistical methodology to overcome problems arising from the quality of the original analyses. They also hoped that combining the results of all trials in a meta-analysis would increase the power to detect realistic treatment differences.[18] The results of the IPD, however, did not refute the results of earlier reviews.

### Methods of the review

All the reviews had protocols that were published some months before publication of the completed review. The protocols included a focused clinical question that pre-stated the patient population, interventions, comparisons and primary and secondary outcomes (PICO) that would be considered in the review and the types of study designs that would be included in the review.[18,19] The reviews included only randomized controlled trials, though one[10] considered quasi-randomized trials as well; such trials that randomize by alternation, hospital numbers, etc are more open to bias than properly randomized trials and their inclusion in meta-analysis can potentially generate unreliable results.[20] Two of the reviews did not clearly state which of the outcomes were primary.[10,11]

### Searching for trials

Since systematic reviews rely largely on identifying, retrieving, evaluating and synthesizing data from other studies, they are prone to selection bias, retrieval bias and publication bias.[21,22] The protocols of the selected reviews also detailed the databases and search strategies that were used to identify relevant trials and overcome these potential sources of bias. All Cochrane collaborative review groups have specialized registers of trials compiled by regular searches of multiple databases such as MEDLINE, EMBASE, CANCERLIT, ‘COCHRANE CENTRAL’, conference proceedings and other sources and these are described in the review group's modules in The Cochrane Library. In addition, review authors searched reference lists of identified trials, contacted authors of trials, manufacturers of drugs and equipments, complementary medicine databases etc, in an attempt to be comprehensive in identifying published and unpublished trials. No language restrictions were used in searching for trials and unpublished trials were also sought and included in order to minimize publication bias; non-English language trials are less likely to be published.[3,14,18] Meta-analyses limited to published trials, compared with those that included both published and unpublished trials, overestimated the treatment effect by an average of 12%.[17] The use of multiple sources also increases the reliability of results as opposed to the use of single sources such as MEDLINE.[21] These searches were run with the help of experienced trial search coordinators in each review group. The Serenoa repens review[14] states, “If our search had been restricted to English language journals we would have missed 13 (62%) trials”.

### Selecting trials and assessing quality

Trials were selected in accordance with the pre-stated inclusion and exclusion criteria. Reasons for exclusion for all excluded trials were reported. Trial selection and quality assessments of included trials were taken by more than one review author, usually independently and disagreements were resolved by discussion, contacting trial authors for more details or by external arbitration. The quality of included trials was assessed using measures that increase the internal validity of a trial.[18] Including trials of poor quality has been shown empirically to produce erroneous results in meta-analyses.[24]

There are four types of biases that occur during the conduct of an RCT that affect internal validity: a) Selection bias: biased allocation to comparison groups, b) Performance bias: unequal provision of care apart from treatment under evaluation, c) Detection bias: biased assessment of outcome and d) Attrition bias: biased occurrence and handling of deviations from protocol and loss to follow-up.[18]

There are four elements considered crucial to handle these biases and improve the internal validity of RCTs.[5,18]

#### 1) Method of generating the random sequence:

The main purpose of randomization is to eliminate selection bias and balance known and unknown confounding factors in order to create a control group that is as similar as possible to the treatment group. Methods for randomly assigning participants to groups, which limits bias, include the use of a table of random numbers and a computer program that generates random numbers. Methods of assignment that are prone to bias include alternating assignment or assignment by date of birth or hospital admission number.

#### 2) Allocation concealment:

A crucial element of the randomization process in preventing selection bias is unpredictability and concealment of the randomization sequence is critical to ensure this. Any method whereby allocation of the next participant is known beforehand, such as alternation or an open list of random numbers, may introduce selection bias. Selection bias in an RCT invalidates the study design and makes the results no more reliable than a non-randomized trial or an observational study. The ideal is central randomization that ensures that generating the randomization sequence and allocating participants are administered by someone who is not responsible for recruiting subjects, such as someone based in a central trial office or pharmacy. Alternatives to central randomization include the use of pre-numbered or coded identical containers of the interventions, which are administered serially to participants; an on-site computer system combined with allocations kept in a locked unreadable computer file; or the use of sequentially numbered, sealed, opaque, envelopes with the allocated interventions described according to the randomization sequence.[18]

Allocation concealment takes place before the interventions are implemented and prevents selection bias that influences all aspects of trial outcomes. Blinding takes place during the interventions and prevents performance and detection bias that may affect only selected outcomes. Adequate allocation concealment is a prerequisite for adequate blinding, prevents selection bias and can always be achieved, whereas blinding takes place during the interventions, may not always be possible and prevents performance and detection bias that may affect only selected outcomes. Empirical research has shown that trials that lack adequate allocation concealment or those that do not report this are associated with bias, often overestimating treatment effects by as much as 41%.[5,20] Concealment of allocation has been found to be more important in preventing bias than other components of trials, such as the generation of the allocation sequence and blinding[5] and is the critical element used to judge the quality of a trial in many Cochrane systematic reviews.[18]

#### 3) Blinding:

Lack of blinding may produce biased reporting of outcomes, especially when subjective outcomes, such as pain or depression scores, are involved, as opposed to objective outcomes such as mortality. A double-blind RCT is a randomized trial in which two groups of individuals (participant and outcome assessor) involved in the trial do not know the identity of the intervention that is given to each participant.[25] Ideally, all properly conducted, so-called, double-blind trials should blind participants, investigators and outcome assessors (be triple-blind trials) to ensure minimal observer bias. Since there is considerable confusion regarding the appropriate use of the term “double blinding”,[26] it is recommended that reports describe who exactly was blinded. Since it is not always possible to maintain blinding in clinical trials where the allocated interventions are difficult to mask adequately (differential side-effects between the interventions), the consolidated standards for reporting trials (CONSORT) statement also recommended that the adequacy of blinding is tested.[27,28]

Adequate blinding has also been shown to be an important attribute of study quality in producing reliable results, particularly when subjective outcomes are used.[5,18]

#### 4) Adequacy of follow-up:

Patients not adhering to treatments in trials generally differ in respects that are related to prognosis.[5] If all participants randomized are not accounted for in the final analysis, attrition bias could result, especially if the number of drop-outs is substantial. The CONSORT statement recommends that authors of papers should state clearly which participants are included in their analyses. The sample size per group or the denominator when proportions are being reported, should be provided for all summary information.[27,28]

‘Intention to treat’ analysis is a strategy in the conduct and analysis of randomized controlled trials that ensures that all patients allocated to either the treatment or control groups are analyzed together as representing that treatment arm, whether or not they received the prescribed treatment or completed the study.[25] This provides conservative estimates of treatment outcomes and if these results do not differ significantly from those produced by an analysis of completers, confidence in the results is strengthened. Because of the variations in trial outcomes and numbers that complete trials, intention to treat is inconsistently associated with biased outcomes.[5]

It is a matter of concern that medical journal editors do not uniformly endorse the requirements of the CONSORT statement in their editorial policies and that uptake of the recommendations has not been uniform in published RCTs leading to considerable loss of information on the quality of trials and often data that could be used in systematic reviews.[29–30]

All Cochrane reviews grade trials according to whether bias was likely to have been present in included trials. Trials where allocation concealment was considered adequate are graded A, those where it is not reported are graded B or unclear and those where it is inadequate are graded C; the last are quasi-randomized trials and are excluded in some reviews. In the selected reviews under consideration, 12/16 included trials in the estrogen review,[10] 5/10 in the review on primary vesicoureteric reflux,[11] all six trials in the bladder cancer review,[12] none of the two trials in the screening for prostatic cancer review,[13] 11/21 in the Serenoa repens review,[14] and one of the two trials in the drug-induced sexual dysfunction review,[15] were graded A for adequacy of allocation concealment.

#### Extracting data

Data were extracted in all reviews by more than one author, working independently. Usually pre-tested data extraction forms are used to ensure that all important aspects of each trial are recorded.

#### Data synthesis

All six reviews were able to quantitatively pool data and perform meta-analysis, though not all Cochrane systematic reviews contain meta-analysis. In performing meta-analysis, for trials that provide data on selected comparisons, e.g. estrogens versus placebo or estrogens versus other interventions, the results of each study are summarized using a measure of effect (such as an odds ratio or a relative risk for dichotomous data or a mean difference for continuous data). For binary measures the data extracted from each trial are the numbers with the outcome of interest in each group, e.g. the number reporting subjective improvement, of the total number randomized in each group. For continuous data, the mean and standard deviation of the outcome measure, e.g. number of pad changes in 24h, as well as the numbers randomized in each group, are extracted. These data from each trial for the comparisons and relevant outcomes are entered into RevMan, the proprietary software of *The Cochrane Collaboration*, and represents the within study comparison of the intervention and control groups. The pooled, weighted measures are combined to produce the summary statistic, usually with confidence intervals that are similar to confidence intervals in single studies. This ensures that participants in each study are only compared to people in the same study, thus preserving the power of randomization.[18]

The individual patient data meta-analysis review on adjuvant chemotherapy for bladder cancer review[12] used the times to event (recurrence, progression or death) for individual patients within trials to calculate the hazard ratios (HR), representing the overall risk of an event for those patients allocated to adjuvant chemotherapy compared with those allocated to no chemotherapy, along with their 95% confidence intervals. Analyses of all endpoints were stratified by trial and the log rank expected number of deaths and variance from each trial used to calculate individual trial hazard ratios and overall pooled hazard ratios (HR) based on the fixed effect model. A hazard ratio is interpreted in a similar way to a risk ratio, as it describes how many times more (or less) likely a participant is to suffer the event at a particular point in time if they receive the experimental rather than the control intervention.[18]

The two RCTs in the antipsychotic drug-induced sexual dysfunction review[16] were crossover trials and these pose special concerns when included in systematic reviews. In a crossover trial all participants receive all interventions in sequence; only the order of interventions is randomly allocated and participants act as their own control. The principal problem associated with crossover trials is that of carry-over in which the effects of an intervention given in one period persist into a subsequent period, thus interfering with the effects of a different subsequent intervention. While both trials in the review used a short-acting drug (sildenafil), one cannot rule out the psychological carry-over effects of success (or failure) with sildenafil (or placebo) into the succeeding treatment period. For meta-analysis of crossover studies, since intervention and control group measures are correlated, paired analyses, accounting for this correlation, are needed and there are statistical methods to accommodate them though they are not entirely adequate. If the crossover design is thought inadequate or if data from paired correlations are not available, then an alternative approach to incorporating crossover trials is to include only data from the first period.[18]

The authors of the Cochrane review[16] chose the option of calculating the standard error of estimates from exactly reported t or p statistics and combing data using the generic inverse variance method. The generic inverse variance method permits the analysis of crossover trials, cluster randomized trials and non-randomized studies, as well as outcome data that are ordinal, time-to-event or rates.[18] However, the authors were cautious in the interpretation of results due to concerns about carry-over effects.

#### Displaying the results of a meta-analysis: The forest plot

**Figure 1:**
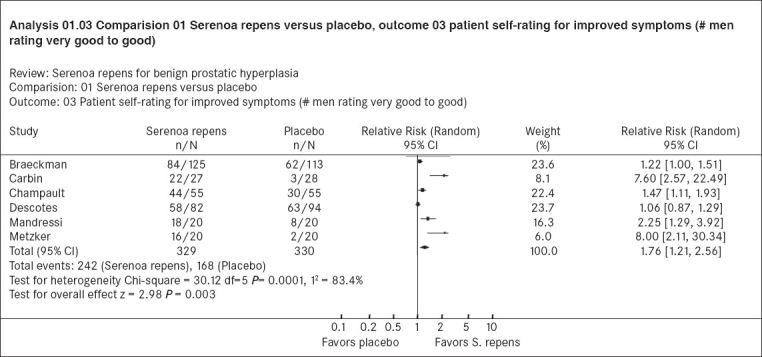
A forest plot from the Cochrane systematic review of Serenoa repens for benign prostatic hyperplasia[14] depicting the relative risk of subjective improvement

A forest plot is a graphical method of displaying the effect estimates from each trial that measures the outcome of a comparison in similar ways in a systematic review. Figure 1 depicts the forest plot of the relative risks and 95% confidence intervals of the six trials that compared Serenoa repens and placebo and that provided data on subjective improvement, from the Cochrane systematic review on Serenoa repens for benign prostatic hyperplasia.[14] A forest plot sometimes resembles a forest of horizontal lines, hence the name. Each of the horizontal lines depicts the 95% confidence intervals for the point estimate (here it is the relative risk) and the squares represent the relative risk of subjective improvement if given Serenoa repens or placebo. The size of the squares represents the sample size of each trial, with larger squares indicating larger study size. The length of the horizontal line depicts the precision of the 95% confidence intervals with smaller lines indicating more precise confidence intervals for the relative risk. The vertical line indicates a relative risk of 1 (no difference between the two interventions). In this analysis, since the outcome is phrased in a positive manner, a relative risk greater than 1 indicates that Serenoa repens was superior to placebo for the outcome and this is indicated by the label at the bottom. In the forest plot, the second, third, fifth and sixth trials had relative risks greater than 1 and the lower and upper limits of the confidence intervals also greater than 1, indicating that in these trials, Serenoa repens was superior to placebo in causing subjective improvement in urinary symptoms. In the first and fourth trial the confidence intervals included 1, indicating uncertainty over the result. None of the trials unequivocally favored placebo. Trials with larger sample sizes and with more people reporting improvement yield more precise results and therefore less variance and are given more weight in the meta-analysis. The diamond at the bottom indicates the pooled, weighted relative risks and confidence intervals of all six trials. Because of the combined sample size of all the trials, the pooled results will always be more precise than the individual trials. Here the pooled estimate (relative risk 1.76) with its 95% confidence intervals (1.2 to 2.56) is greater than 1, indicating that treatment with Serenoa repens in men with benign prostatic hyperplasia is likely to increase the chances of subjective improvement in urinary symptoms by 76%, but this might be as little as 2% or as great as 156%. Since these do not include a relative risk of 1, this automatically indicates a statistically significant difference (*P*< 0.05; in this instance the exact *P* value is 0.003, shown at the bottom of the graph). The forest plot provides a simple visual representation of the amount of variation between the results of the studies, as well as an estimate of the overall result of all the studies together.

**Figure 2:**
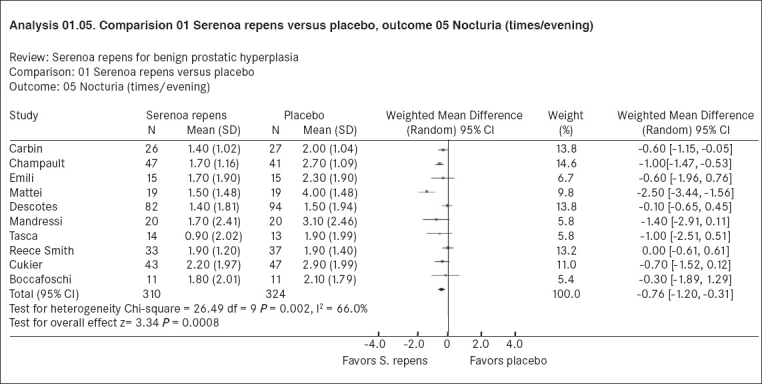
A forest plot from the Cochrane systematic review of Serenoa repens for benign prostatic hyperplasia[14] depicting the mean difference in the frequency of nocturia

Figure 2 from the same review[14] shows the results for 10 trials that measured the frequency of nocturia on Serenoa repens or placebo. Here the effect estimate is the mean difference in frequency of nocturia between interventions, with weights applied to each trial by RevMan as the inverse of the variance, as in the previous example. The pooled weighted mean difference from all 10 trials again favored Serenoa repens.

#### Assessing heterogeneity

One of the main concerns about meta-analysis is whether the adding together of trials done in different parts of the world and under different conditions of care would yield meaningful results. This variability in results (heterogeneity) can arise due to various reasons. Clinical heterogeneity refers to the differences in studies due to differences in participant characteristics and interventions. Methodological heterogeneity arises from differences in trial design and methodology. Statistical heterogeneity refers to incompatibility in the statistical results. The former two may lead to the latter and requires the review authors to combine trial results only if there are no important clinical differences in the study characteristics or trial methods.

There are three ways of evaluating for statistical heterogeneity. One way of doing this is to look at a graphical display of the results. If the confidence intervals for the results of each study do not overlap, it suggests that heterogeneity is present. In Figures 1 and 2, there are trials whose confidence intervals do not overlap.

The other method of evaluating statistical heterogeneity is to do a Chi-square test for homogeneity (also called Cochran's Q). As a rule of thumb, the Chi-square statistic has a value equal to its degrees of freedom (one less than the number of trials); values larger than the degrees of freedom would give smaller *P* values and indicate significant statistical heterogeneity. In Figure 1, the Chi-square value is 30.12 with six trials; in Figure 2 the Chi-square value is 26.49 with 10 trials. In both instances, these indicate the possibility of significant heterogeneity and confirmation that this variability is statistically significant comes from *P* values of 0.0001 and 0.002 respectively. When there is statistically significant heterogeneity, it suggests that the observed differences in results between trials are likely to be caused by factors other than random error (chance).[18]

The Chi-square test has low power in the (not uncommon) situation of a meta-analysis when trials have small sample size or are few in number. Thus a *P* value of 0.10, rather than the conventional level of 0.05, is used to determine statistical significance. A further problem with the test is that when there are many studies in a meta-analysis, the test has a high power to detect a small amount of heterogeneity that may be clinically unimportant.

Since it is likely that combining trials will inevitably give rise to some amount of heterogeneity, methods have been developed for quantifying inconsistency across studies that move the focus away from testing whether heterogeneity is present to assessing its impact on the meta-analysis. A useful statistic for quantifying inconsistency is I^2^ (derived from Cochran's Q where I^2^ [(Q - df)/Q] × 100%).[31] This describes the percentage of the variability in effect estimates that is due to heterogeneity rather than sampling error (chance). A value less than 25% suggests little heterogeneity, while a value greater than 50% may be considered substantial heterogeneity.[18,31] The I^2^ in Figures 1 (83.4%) and 2 (66%) are greater than 50% indicating substantial heterogeneity in both meta-analyses.

In the presence of significant heterogeneity, some authors chose not to combine the results in a meta-analysis, especially if the variability is due to clinical or methodological differences in trials. If important differences can be identified in one or two trials that have non-overlapping confidence intervals, then the impact of removing these trials on the overall results can be tested in a sensitivity analysis. In other instances, the effects of sub-grouping trials by some prognostic variable (such as size of the prostate) can be evaluated to see if pooled results differ and variability is reduced by this sub-grouping.

When no important differences can be readily identified that explain the source of heterogeneity, a cautious interpretation of the results is warranted. The use of a random effects model (that assumes that the pooled effect is influenced by inter-trial variability as well as random error) as opposed to a fixed effects model (that does not account for significant inter-trial variability) in the meta-analysis provides more conservative estimates of overall effects (since the former assigns more equally between trials).

One can experiment with this online with the Serenoa repens systematic review[14] in The Cochrane Library (try it). In the analyses section of the review, if the relevant comparison and outcomes (Analysis 1; comparison 01; outcome 03) are selected, one can adjust the statistical analysis to shift from random effects to fixed effects. The pooled estimates for Figure 1 with the random effects model yields a Relative Risk of 1.76 and 95% confidence intervals of 1.21 to 2.56. The fixed effects model changes the Relative Risk to 1.45 and the 95% confidence intervals to 1.28 to 1.65; the confidence intervals are more precise. The indicators of heterogeneity (I^2^ 83.4%) do not change, however, and the results should still be interpreted with caution.

#### Interpreting results

In the review on ‘interventions for primary vesicoureteral reflux’[11] (VUR), the authors found that the, “Risk of UTI by 1-2 and 5 years was not significantly different between surgical and medical groups (by 2 years RR 1.07, 95% CI 0.55 to 2.09; by 5 years RR 0.99; 95% CI 0.79 to 1.26). Combined treatment resulted in a 60% reduction in febrile UTI by 5 years (RR 0.43, 95% CI 0.27 to 0.70) but no concomitant significant reduction in risk of new or progressive renal damage at 5 years (RR 1.05, 95% CI 0.85 to 1.29)”. This review included 10 trials involving 964 children comparing long-term antibiotics and surgical correction of VUR with antibiotics (seven trials), antibiotics with no treatment (one trial) and different materials for endoscopic correction of vesicoureteral reflux (two trials). The authors concluded that “It is uncertain whether the identification and treatment of children with VUR confers clinically important benefit. The additional benefit of surgery over antibiotics alone is small at best.” By acknowledging uncertainty in the presence of wide confidence intervals that included a Relative Risk of 1, the authors did not mistake ‘no evidence of effect’ with ‘evidence of no effect‘. The authors also reported the number needed to treat when they concluded that, “Assuming a UTI rate of 20% for children with VUR on antibiotics for five years, nine re-implantations would be required to prevent one febrile UTI, with no reduction in the number of children developing any UTI or renal damage.”

Cochrane reviews provide the option for readers to comment online on any review and the authors of this review[11] responded to a reader's comment that expressed surprise that surgery does not help reflux by reiterating that the results of the review did not suggest any benefit for surgery versus conservative management, irrespective of the grade of reflux, except for the frequency of febrile episodes of urinary tract infection.

The Cochrane review on screening for prostatic cancer[13] included two randomized controlled trials with a total of 55,512 participants. The authors re-analyzed data using ‘intention-to-screen’ in their meta-analysis. This conservative analysis showed no statistically significant difference in prostate cancer mortality between men randomized for prostate cancer screening and controls (RR 1.01, 95% CI: 0.80-1.29), though screening did increase cancer detection rate. The authors are awaiting the results of two ongoing trials to make more firm conclusions regarding the value of screening.

The predictive ability of meta-analyses of RCTs have been questioned by discrepancies, mainly in relation to the size of the effect rather than the direction, detected between published meta-analyses and subsequent large randomized controlled trials on the same topic.[32] The review on Serenoa repens for benign prostatic hyperplasia[14] was first published in 1998 and updated in 2002. It showed from 21 randomized trials of 3139 men, lasting four to 48 weeks, that Serenoa repens provided mild to moderate improvement in urinary symptoms and flow measures compared to placebo and that it produced similar improvement in urinary symptoms and flow compared to finasteride but was associated with fewer adverse effects. A subsequent RCT[15] assigned 225 men over the age of 49 years who had moderate-to-severe symptoms of benign prostatic hyperplasia to one year of treatment with saw palmetto extract (160 mg twice a day) or placebo. There was no significant differences between the saw palmetto (serenoa repens) and placebo groups in the change in the American Urological Association Symptom Index (AUASI) scores, maximal urinary flow rate, prostate size, residual volume after voiding, quality of life or serum prostate-specific antigen levels during the one-year study.

The reasons for this apparent discrepancy in results are not hard to find. The RCT followed up participants for a year while the mean duration of follow-up in the review was 13 weeks (range 4-48 weeks). The main outcome measures in the RCT were the AUASI scores and the maximal urinary flow rate. In the review, the main outcomes were change in urological symptom scale scores or global report of symptoms. In the comparison of Serena repens with placebo, only two small trials provided such scores using two different scales; these were not combined since one reported on endpoint scores and the other on change from baseline. Both showed inconsistent results. It would be possible to combine the results from the RCT with the scores from the two included trials in the review using the standardized mean difference (SMD) instead of the weighted mean difference (WMD). The SMD divides the mean outcome score between groups by the standard deviation of outcome among participants and enables continuous data from different measures examining similar outcomes to be combined.[18] In the review, peak urinary flow rates from nine trials including 723 participants favored Serenoa repens. If the data for peak flow rates from the RCT were pooled with the existing data from the review one would be able to assess whether the pooled results would still favor Serenoa repens. The review is now out of date and one would expect that an update will include the results of this and other trials published since 2002.

The results of the saw palmetto trial indicate uncertainty of effect and not ‘evidence of no effect’. Combining these results with those in the Cochrane systematic review will provide updated and reliable evidence that can be incorporated into one's clinical expertise and the wishes of ones’ patients in the endeavor called evidence-based urology.

## CONCLUSIONS

Progress in science is cumulative. *The Cochrane Library* provides a useful source of the cumulated evidence from good quality randomized controlled clinical trials in the form of updated systematic reviews. Application of this evidence to clinical practice requires clinicians to assess whether the characteristics of the included trials resemble their clinical conditions and patient characteristics. When evidence is scant, even from systematic reviews, then conducting pragmatic trials may offer the only solution on which to base one's practice on reliable and relevant evidence, rather than on opinion or conjecture. *The South Asian Cochrane Network *(www.cochrane-sacn.org) provides training workshops and mentoring support across the region to those interested in undertaking systematic reviews of relevance to healthcare in the region; urologists are especially welcome.

## References

[1] Sackett D (2000). Evidence-based medicine: How to practice and teach EBM.

[2] Concato J, Shah N, Horwitz RI (2000). Randomized, controlled trials, observational studies, and the hierarchy of research designs. N Engl J Med.

[3] Pai M, McCulloch M, Gorman JD, Pai N, Enanoria W, Kennedy G (2004). Systematic reviews and meta-analyses: An illustrated, step-by-step guide. Natl Med J India.

[4] Mulrow CD (1994). Rationale for systematic reviews. BMJ.

[5] Jüni P, Altman DG, Egger M (2001). Systematic reviews in health care: Assessing the quality of controlled clinical trials. BMJ.

[6] Tharyan P (2005). The Cochrane Schizophrenia Group: Preparing, maintaining and disseminating the evidence for interventions used for people with schizophrenia. Int Rev Psychiatry.

[7] Evans D (2003). Hierarchy of evidence: A framework for ranking evidence evaluating healthcare interventions. J Clin Nurs.

[8] Jadad AR, Cook DJ, Jones A, Klassen TP, Tugwell P, Moher M (1998). Methodology and reports of systematic reviews and meta-analyses: A comparison of Cochrane reviews with articles published in paper-based journals. JAMA.

[9] Jørgensen AW, Hilden J, Gøtzsche PC (2006). Cochrane reviews compared with industry supported meta-analyses and other meta-analyses of the same drugs: Systematic review. BMJ.

[10] Moehrer B, Hextall A, Jackson S (2003). Oestrogens for urinary incontinence in women. Cochrane Database Syst Rev.

[11] Wheeler DM, Vimalachandra D, Hodson EM, Smith GH, Craig JC (2004). Interventions for primary vesicoureteric reflux. Cochrane Database Syst Rev.

[12] Advanced Bladder Cancer (ABC) Meta-analysis Collaboration (2006). Adjuvant chemotherapy for invasive bladder cancer (individual patient data). Cochrane Database Syst Rev.

[13] Ilic D, O'Connor D, Green S, Wilt T (2006). Screening for prostate cancer. Cochrane Database Syst Rev.

[14] Wilt T, Ishani A, Mac Donald R (2002). Serenoa repens for benign prostatic hyperplasia. Cochrane Database Syst Rev.

[15] Bent S, Kane C, Shinohara K, Neuhaus J, Hudes ES, Goldberg H (2006). Saw palmetto for benign prostatic hyperplasia. N Engl J Med.

[16] Berner MM, Hagen M, Kriston L (2007). Management of sexual dysfunction due to antipsychotic drug therapy. Cochrane Database Syst Rev.

[17] Moher D, Cook DJ, Eastwood S, Olkin I, Rennie D, Stroup DF (1999). Improving the quality of reports of meta-analyses of randomised controlled trials: The QUOROM statement. Quality of Reporting of Meta-analyses. Lancet.

[18] Higgins JP, Green S (2005). Cochrane handbook for systematic reviews of interventions 4.2.5 [updated May 2005]. The Cochrane Library, Issue 3.

[19] Richardson WS, Wilson MC, Nishikawa J, Hayward RS (1995). The well-built clinical question: A key to evidence-based decisions. ACP J Club.

[20] Schulz KF, Chalmers I, Hayes RJ, Altman D (1995). Empirical evidence of bias. Dimensions of methodological quality associated with estimates of treatment effects in controlled clinical trials. JAMA.

[21] Dickersin K (1990). The existence of publication bias and risk factors for its occurrence. JAMA.

[22] Felson DT (1992). Bias in meta-analytic research. J Clin Epidemol.

[23] Suarez-Almazor ME, Belseck E, Homik J, Dorgan M, Ramos-Remus C (2000). Identifying clinical trials in the medical literature with electronic databases: MEDLINE alone is not enough. Control Clin Trials.

[24] Moher D, Pham B, Jones A, Cook DJ, Jadad AR, Moher M (1998). Does the quality of reports of randomized trials affect estimates of intervention efficacy reported in meta-analyses?. Lancet.

[25] Jadad AR (1998). Randomized controlled trials: A user's guide.

[26] Devereaux PJ, Manns BJ, Ghali WA, Quan H, Lacchetti C, Moutori VM (2001). Physician interpretations and textbook definitions of blinding terminology in randomized controlled trials. JAMA.

[27] Begg CB, Cho MK, Eastwood S, Horton R, Moher D, Olkin I (1996). Improving the quality of reporting of randomized controlled trials: The CONSORT statement. JAMA.

[28] Altman DG, Schulz KF, Moher D, Egger M, Davidoff F, Elbourne D (2001). The revised CONSORT statement for reporting randomized trials: Explanation and elaboration. Ann Intern Med.

[29] Altman DG (2005). Endorsement of the CONSORT statement by high impact medical journals: Survey of instructions for authors. BMJ.

[30] Hewitt C, Hahn S, Torgerson DJ, Watson J, Bland JM (2005). Adequacy and reporting of allocation concealment: Review of recent trials published in four general medical journals. BMJ.

[31] Higgins JP, Thompson SG, Deeks JJ, Altman DG (2003). Measuring inconsistency in meta-analyses. BMJ.

[32] LeLorier J, Gregoire G, Benhaddad A, Lapierre J, Derderian F (1997). Discrepancies between meta-analyses and subsequent large randomized, controlled trials. N Engl J Med.

